# Low Renalase Levels in Newly Diagnosed CML: Dysregulation Sensitive to Modulation by Tyrosine Kinase Inhibitors

**DOI:** 10.3390/pathophysiology31040053

**Published:** 2024-12-10

**Authors:** Jelena Milenkovic, Dijana Stojanovic, Sanja Velickovic, Branka Djordjevic, Goran Marjanovic, Maja Milojkovic

**Affiliations:** 1 Department of Pathophysiology, University of Nis, Faculty of Medicine, 18000 Nis, Serbia; dijana.stojanovic@medfak.ni.ac.rs (D.S.); maja.milojkovic@medfak.ni.ac.rs (M.M.); 2 Clinic of Hematology, Allergology and Clinical Immunology, University Clinical Center in Nis, 18000 Nis, Serbia; sanjavelickovicdr@gmail.com (S.V.); goran.marjanovic@medfak.ni.ac.rs (G.M.); 3 Department of Biochemistry, University of Nis, Faculty of Medicine, 18000 Nis, Serbia; branka.djordjevic@medfak.ni.ac.rs

**Keywords:** myeloproliferative neoplasms, proinflammatory cytokines, tyrosine kinase inhibitors, signal transducer and activator of transcription 3 (STAT3)

## Abstract

**Background:** A dysregulated proinflammatory microenvironment is considered one of the reasons why current therapies of chronic myeloid leukemia (CML) with tyrosine kinase inhibitors (TKI) do not secure disease control. Therefore, the development of BCR-ABL1-independent therapies is encouraged. Renalase (RNLS) is a multifunctional protein that exhibits both enzymatic and non-enzymatic cytokine-like properties, along with potent anti-inflammatory and anti-apoptotic effects. It is expressed in various tissues, including tumors. **Methods:** We investigated the levels of RNLS in the blood of CML patients in the chronic phase, treatment naïve patients, and those in remission under TKI treatment (either imatinib or nilotinib) and compared them to healthy individuals. **Results:** Renalase concentration was markedly decreased in treatment-naive CML patients compared to other groups (*p* = 0.000), while lower levels in the TKI group were not statistically significant compared to controls. The levels correlated negatively with the total leukocyte and neutrophil count (*p* < 0.05), while a positive correlation was present with CRP levels in treatment naïve patients. **Conclusions:** Dynamic regulation of RNLS expression and activity is coupled with transcription factors NF-κB and STAT3. Interpretation of our results might rely on differential requirements of activated STATs (STAT3/5) during CML clone development and maintenance, including the observation of RNLS rise upon TKI introduction. Overall, our research provides new insights into the field of hematological malignancies. Unlike other malignancies studied, RNLS plasma levels are significantly decreased in CML. In future perspectives, RNLS could potentially serve as a diagnostic, prognostic, or therapeutic option for these patients.

## 1. Introduction

A recent and developing concept perceives myeloproliferative neoplasms (MPNs) as a biological continuum of inflammation-related cancers with a prominent role of chronic inflammation in the modulation of disease phenotype and supporting its progression [1,2,3,4,5]. Constitutively upregulated BCR-ABL1 fusion tyrosine kinase (TK) activity in chronic myeloid leukemia (CML) initiates complex signaling that dysregulates and transforms hematopoietic stem cells into CML (leukemia) stem cells or LSC. Several pathways are initialized and further sustained by BCR-ABL1 kinase, including Janus kinase (JAK)/signal transducer and activator of transcription (STATs), phosphoinositide 3-kinase (PI3K)/Akt/mammalian target of rapamycin (mTOR), mitogen-activated protein kinases (MAPK), extracellular-signal-regulated kinase (ERK), etc. However, the introduction of TK inhibitors (TKI) has led to a major advancement in CML treatment [6,7].

Nevertheless, accumulating evidence points to the MPNs as pan-hematopoietic diseases due to the substantial contribution of the local non-malignant bone marrow stromal compartments, which display several abnormalities that support cytokine overproduction and favor malignant over normal hematopoiesis. A key mediator of this onco-inflammation is the nuclear factor kappa B (NF-κB), coupled with a marked proinflammatory milieu involving tumor necrosis factor (TNF)-α, interleukin (IL)-1β, IL-6, and other cytokines, chemokines, and growth factors. The dysregulated microenvironment is considered one of the main reasons why current TKI therapies do not secure complete control of the disease [1,2,8,9,10,11,12]. Given the persistence of malignant clones and continuously elevated cytokines, despite the treatment, new therapeutic strategies that aim at targeting BCR-ABL independent networks in MPNs are repeatedly encouraged [1,13,14].

Renalase (RNLS) is a multifunctional protein, synthesized and secreted by multiple cell types. At first, it was recognized as a soluble monoamine oxidase, produced by renal tubulocytes upon stimulation of α-adrenoceptor/NF-κB pathways and related to inactivation of catecholamines. In the following years, RNLS was characterized as a cytokine-like molecule that binds (via its RP-220 domain) to plasma membrane receptor Ca2+-ATPase 4b (PMCA4b) and exhibits profound effects on cellular cycle and survival by activating several signaling pathways [13,15,16,17,18]. Contrastingly, intracellular RNLS affects energy metabolism as an oxidoreductase that transforms isomeric β-NAD(P)H forms back to β-NAD(P)+, thereby impeding their negative effect on primary metabolism dehydrogenases and mitigating mitochondrial dysfunction [14,16,17,18,19,20]. Subsequently, RNLS was identified in several different tissues (e.g., heart, liver, small intestines, nervous system, adipose tissue, skeletal muscles, placenta, etc.) (reviewed in [16]), as well as tumor cells and tumor-associated macrophages [13,21,22,23,24].

Renalase provides potent anti-inflammatory and anti-apoptotic effects, mediating resistance to stressful stimuli and cellular injury. It evokes p38 kinase MAPK, NF-κB, and JAK/STAT signaling, the PI3K/AKT pathway, and antiapoptotic B cell lymphoma (BCL) 2 pathways. It inhibits the c-Jun N-terminal kinase (JNK) MAPK, while the ERK 1/2/MAPK pathway is differentially affected [13,14,15,16,17,18,24,25,26]. Moreover, RNLS activation interferes with oxidative stress, hinders fibroproliferative response, affects autophagy, and supports cytoprotective effects of sirtuins [14,16,17,18,19,20].

Recent research indicates that RNLS expression is higher in certain cancers (pancreatic, breast, melanoma, etc.) than in benign tissues. Its plasma concentration correlated with the clinical presentation of pancreatic adenocarcinoma, while an inverse correlation with patients’ survival was observed, suggesting a pathogenic role of RNLS in these malignancies [18,22,23,24,25]. Conversely, other reports present low RNLS plasma concentrations in various pathologies, such as those seen in patients with resistant hypertension, renal insufficiency, pre-eclampsia, and schizophrenia ([13], reviewed in [17]), and with impaired immune/inflammatory response in severe COVID-19 [17,25]. This might not be surprising given the dynamic regulation of RNLS expression and activity coupled with impacts from endocrine and immune systems [17,19].

To our knowledge, RNLS was not evaluated in hematologic malignancies. Considering the abovementioned pathways that are dysregulated in CML, while they are affected by RNLS, we believe RNLS is an interesting research object, both as a target molecule and a serological biomarker of the disease. Therefore, our main aim was to explore RNLS in patients with CML in the chronic phase, treatment naïve patients, and patients undergoing TKI therapy in comparison to healthy individuals as controls.

We present our findings of significantly decreased RNLS plasma levels in patients with CML compared to controls, as well as lower levels in treatment naïve than patients taking TKI, suggesting RNLS alteration is a part of leukemic transformation in these individuals, but which seems to be sensitive to modulation by TKI.

## 2. Materials and Methods

This prospective cross-sectional case-control investigation included 75 consecutive adult patients diagnosed with CML in stable chronic-phase disease. There are two groups of patients: (1) a group with 55 patients already taking TKI therapy for at least 6 months and who are in hematological and cytogenetic remission, and (2) a group of 20 newly diagnosed CML treatment naïve patients. All patients were recruited at the Clinic of Hematology, Allergology and Clinical Immunology, University Clinical Center in Nis, Serbia, from January 2023 to July 2024.

Clinical, molecular, and biochemical information was prospectively and retrospectively obtained from patients’ medical history. The patients were diagnosed according to the current WHO classification and are monitored by hematological and cytological assessments and measurement of BCR-ABL1 transcript levels using real-time quantitative polymerase chain reaction standardized to the international reporting scale. Patient risk stratification was calculated using the Sokal prognostic score [3,27].

The exclusion criteria included the presence of arterial hypertension, heart disease, kidney disease, liver dysfunction, acute inflammatory conditions, other malignancies, stroke, and unwillingness to participate in the study. None of the patients were actively using corticosteroid therapy. The control group comprised 20 healthy community-based adult volunteers, not taking any medications, and who were age-matched to the patients. All subjects gave and signed an informed consent before participating in the study.

The collected EDTA blood samples were analyzed at the Faculty of Medicine, University of Nis, Serbia. Plasma samples were safely stored at −80 °C until a final assessment of biomarkers using quantitative sandwich enzyme-linked immunoassay (ELISA) technique was conducted, using the manufacturer’s protocol. Levels of RNLS were measured by the ELISA method using the FineTest^®^ Human RNLS (Renalase) ELISA Kit (EH1250) (Wuhan Fine Biotech Co., Ltd., Wuhan, Hubei, China), with a range of detection between 0.781–50 ng/mL, whereas the minimum detectable level of RNLS was 0.469 ng/mL. The assay precision was reported as the coefficient of variation (%CV), including intra-assay %CV of 5.82 and inter-assay %CV of 4.82.

Complete blood count (CBC) parameters (number of erythrocytes, leukocytes with differential count, platelets, and hemoglobin concentrations) and C-reactive protein (CRP) concentration were assessed using the COULTER^®^ AcT Diff Analyzer (Beckman Coulter Corporation, Hialeah, FL, USA).

The work has been carried out according to the World Medical Association Code of Ethics (Declaration of Helsinki) for experiments on human subjects. The study was approved by the Faculty’s Ethical Board (Decision No. 12-11238/2-2 from 12.10.2022) of the Faculty of Medicine University of Nis, Serbia.

Statistical analysis was carried out using SPSS 25.0 software (SPSS Inc, Chicago, IL, USA). Data are presented as percentages and mean ± standard deviation (SD) or median ± interquartile range (IQR), based on the normal distribution of the samples (Shapiro–Wilk test). One-way analysis of variance (ANOVA) was applied to compare the RNLS blood concentrations between the groups, and the post-hoc Dunnett T3 test for pairwise comparison was used to determine a difference for each pair when the variances are unequal (95% confidence interval, CI). In addition, the Mann–Whitney U test or Student’s *T*-test was used to compare differences between two independent groups. The Pearson correlation coefficient test was also checked. Significance was assumed at a value of *p* < 0.05.

## 3. Results

The patients’ mean age was 58.3 ± 13.25 years for patients and 55.7 ± 11.08 for healthy controls (*p* > 0.05). There were 46.7% males and 53.3% females in the patient group. The age at diagnosis of CML was 68.5 ± 13.4 years, with the median disease duration of 5.82 ± 3.42 years. As expected, the white blood count (WBC), neutrophil, and platelet counts were higher in patients than in controls. Baseline characteristics of the study’s participants with CBC parameters are shown in Table 1.

Patients were under treatment with TKI, imatinib, or nilotinib, and in complete hematological and cytogenetic remission with the major molecular response (MMR or MR3, BCR-ABL1 values of ≤0.1% IS) present in 80% (n = 44) at the last control. Results were not evaluated according to the BCR-ABL transcript levels of ≤0.01% IS (deep molecular response) due to the lack of data in some patients. About half received imatinib (45.5%), 400 mg once daily, or nilotinib (54.5%), 300 mg twice daily. Nilotinib is introduced to those who have lost a therapeutic response (complete cytogenetic response) to imatinib, and this was rarer when there was no primary effect of imatinib. The average duration of TKI treatment was 41.2 ± 31.3 months.

**Figure 1:**
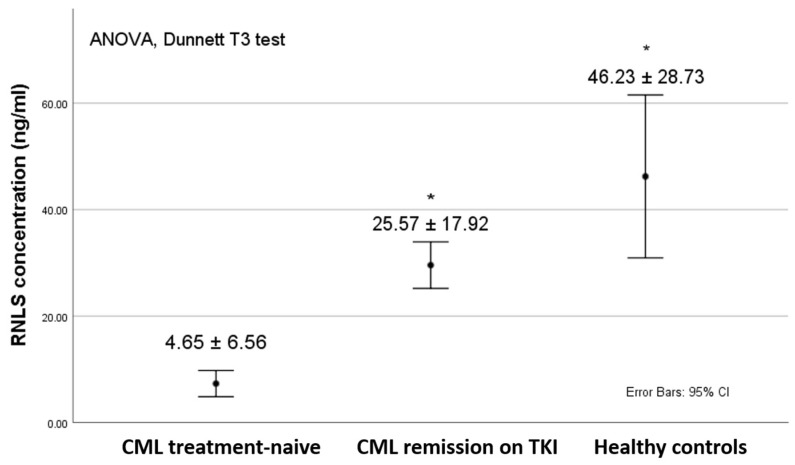
Comparison of the RNLS concentration between the groups. * *p* = 0.000 compared to the CML treatment-naïve group.

We determined a statistically significant difference between the mean levels of RNLS between our groups using the ANOVA test (*p* = 0.000, F = 22.419). Post hoc Dunnett’s T3 showed a significant difference between newly diagnosed (treatment naïve) CML patients (4.65 ± 6.56) and healthy controls (46.23 ± 28.73) as well as compared to the patients taking TKI (25.57 ± 17.92) for *p* = 0.000. There was no marked difference between patients on TKI treatment and the controls (Figure 1).

There was no significant difference in the RNLS plasma concentration between patients who received imatinib or nilotinib (24.36 ± 8.73 vs. 26.61 ± 18.09 ng/mL, *p* = 0.617). We did not determine a significant difference between RNLS levels according to the Sokal score.

**Figure 2:**
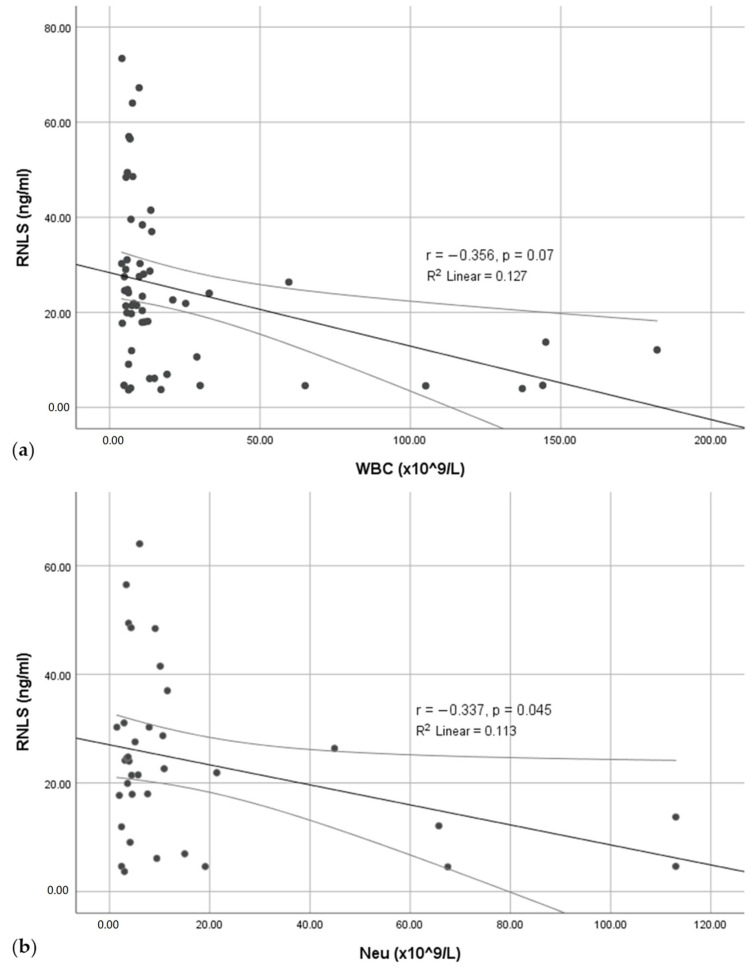
Correlation between the RNLS concentration and leukocyte count. (**a**) The correlation of RNLS to white blood cell (WBC) count; (**b**) The correlation of RNLS to neutrophil granulocyte count.

A significant negative, but weak, correlation was present between the RNLS concentration and total leukocyte count and neutrophil count in the blood (*p* = 0.007, r = −0.356 and *p* = 0.045, r = −0.337, respectively) (Figure 2).

As expected, values of RNLS to WBC ratio were significantly lower in treatment naïve patients (0.346 ± 0.348) than in patients on TKI (4.024 ± 3.324) and healthy subjects (6.304 ± 3.224), though there was no marked difference between the last two groups (One-way ANOVA; *p* = 0.000, F = 12.307).

Although around the upper limit of the reference range, the patient’s CRP levels were significantly higher compared to controls but without marked differences between the groups (Table 1). Still, only the values in treatment naïve CML patients correlated significantly and positively with RNLS concentration (*p* = 0.008, r = 0.620).

## 4. Discussion

Although the incidence of CML progression has dramatically decreased with currently used TKI, they are incapable of permanently depleting malignant clones, and patients relapse after early success. Genetic instability of the malignant clone predisposes it to additional mutations (e.g., in TP53, MYC genes), which, in combination with extrinsic modulation from the microenvironment, will support disease progression and TKI resistance [6,7,8,9,15,28,29]. Patients with CML show increased levels of circulating proinflammatory cytokines, and a cytokine-directed intervention is one of the trusted perspectives in disease management [1,3,8,9,10,11,15,29]. This is particularly advantageous as it addresses BCR-ABL1 independent upregulation of several signaling routes that maintain malignancy, such as the PIK3/Akt/mTOR pathway or STAT3 activation [7,10].

Renalase is a pleiotropic, cytokine-like molecule capable of triggering multiple downstream signals that engage in pro-survival, anti-inflammatory, antioxidant, and anti-fibroproliferative actions. Thus, it is designated as a survival factor important for cellular response to injury, although its function has not been fully elucidated. Renalase gene expression is controlled by several transcription factors, including NF-κB and hypoxia-inducible factor (HIF)-1α, Sp1, and ZBP89, while STAT3 is an enhancer of its gene expression [25,30]. Tumor necrosis factor α and reactive oxidative species (ROS) upregulate RNLS expression via NF-κB signaling, while similar signaling mediation is observed for RNLS and IL-6 [9,18,19,24,31].

The latest research on COVID-19 pointed out a beneficial upregulation of RNLS that aimed at counteracting inflammation and oxidative stress in patients. Its dysregulation was reflected in decreased blood concentration and was associated with a severe clinical picture, worse outcomes, and higher mortality. Patients with low RNLS/high IL-6 had the worst prognosis. The RNLS rise depended on the immune system and appeared to attenuate proinflammatory cytokines production [18,26,32].

Our results show markedly decreased RNLS plasma levels in newly diagnosed, treatment naïve patients with chronic phase CML, compared to healthy individuals as well as patients taking BCR-ABL1 kinase inhibitors. The RNLS concentration was also lower in those on TKI than in controls, but not statistically significant. The levels negatively correlated with the WBC count, which was similar to the results of the aforementioned study of COVID-19. An additional negative correlation existed with the neutrophil count, the cells that make a substantial part of the CML clone. Renalase positively correlated with CRP in newly diagnosed CML patients, reiterating its relationship with inflammation and modulatory effects of TKI. Several studies report RNLS association with markers of inflammation but without consistency, that is, while being positively correlated with CRP in peritoneal dialysis patients, it was negatively correlated with ferritin, WBC, and IL-1 values in COVID-19 [27,33].

The low RNLS levels were somewhat surprising given the enhanced activity of its inflammatory stimuli, high ROS production (BCR-ABL1-induced via PI3K/Akt, STAT1, and STAT5), and evidence of increased RNLS levels (blood and tissue) in several other malignancies [7,19,24,34,35]. A question is whether the decreased RNLS level is a result of the oncogene-directed transformation, or a secondary consequence of local signaling (mal)adaptations, in transformed or neighboring cells.

Interpretation of our findings might partially rely on differential requirements of activated STATs during the initiation of BCR-ABL1 transformation and the maintenance of leukemic cells. JAK/STAT signaling is one of the key downstream pathways in BCR-ABL1-positive cells and is led by STAT5 and STAT3 (RNLS enhancer). Both STATs are necessary for the initial step, but later on, maintenance of LSCs demands only STAT5 persistence [10,36], and constantly high levels of this transcription factor were shown in the nuclei of malignant cells [37,38]. Functional redundancy between STAT3 and STAT5 with competitive status in the regulation of certain genes is an additional possibility to consider, as demonstrated for BCL6 protein in several cell types [37,39].

The differential status of STAT3/5 also offers an interpretation of the RNLS rise upon TKI introduction. Namely, in imatinib-treated CML patients, there is an upregulation of interferon γ (IFNγ) production by activated T cells, which leads to the inhibition of the constitutive STAT5 activation. At the same time, STAT3 is increasingly operative, but through an extrinsically driven (stroma-derived, oncogene-independent) induction of CML cells [34,40,41]. Recent work identified this JAK1-STAT3-activating signaling and IL-6 as responsible for TKI resistance, thus proposing novel, even potentially curative, therapeutic targets [15].

Another perplexity concerns an aspect of CML LSCs’ energetic metabolism and its potential reflection on RNLS. Leukemic cells undergo substantial metabolic derangements, with oxidative phosphorylation and aerobic glycolysis being both demonstrated. The chronic-phase CML LSCs display an oxidative phenotype compared to non-leukemic hematopoietic stem cells [42]. As reported, the NAD-dependent deacetylase sirtuin 1 is overexpressed in primitive as well as persistent LSCs and can upregulate oxidative phosphorylation by enhancing transcription of mitochondrial electron transport chain–related genes [43]. Renalase can affect mitochondrial function by increasing cellular NAD+ levels, thereby assisting the activity of sirtuins 1 and 3, and supporting the maintenance of oxidative energy metabolism [16,19,20].

Nevertheless, a recent study argues that TKI-persistent CML LSCs are transcriptionally different, with a distinct proteome suggestive of metabolic changes associated with marked upregulation of proteins contributing to glycolysis, HIF, and the forkhead box O (FOXO) signaling. As aforementioned, active STAT3 signaling provides an important contribution to promoting these metabolic changes. Unlike untreated LSCs, the majority of TKI-persistent LSCs had activated the pTyrSTAT3 pathway that suppresses oxidative phosphorylation and dysregulates mitochondrial metabolism, ultimately pushing the persistent cells to depend on aerobic glycolysis (where NADH is oxidized to NAD+) [15,40,44]. In line with this, RNLS could be purposely activated via STAT3 and/or HIF as a response to stress conditions, aiming to control inflammation, redox balance, and metabolic disturbances.

A tempting concept involves the relationship between RNLS and lipid metabolism. Specifically, in a steatotic liver model of mice fed with a high-fat diet, the findings of reduced liver RNLS expression and blood levels were attributed to a decreased STAT3 expression, possibly through enhancement of its suppressors of cytokine signaling (SOCS3) regulator. On the other hand, LSCs can exploit the adipocyte-rich niche by inducing a metabolic alteration in adipocytes with enhanced lipolysis. Among others, TNF-α, IL-1α, and IL-1β were capable of inducing lipolysis in this setting with a much higher release of free fatty acids that are then utilized by the CD36+ LSC subpopulation [31,44,45,46].

An interesting study emphasized the protective role of RNLS deletion against immune response in a model of autoimmune diabetes [47]. The study was based on the notion that tumor metabolism can influence local immunity and contribute to tumor evasion. The lack of RNLS in pancreatic β-cells led to perturbations in their metabolism that further induced a broad metabolic modification and a change in the transcriptome of graft-infiltrating leukocytes. Of note, antigen-presenting cells in RNLS-deficient grafts had higher PD-L1 expression, which turned out to be a deciding factor in suppressing immune-mediated killing. The loss of RNLS in β-cells impacted immune cell metabolism with a marked increase in glycolysis and enhanced lipid metabolism that affected their function favoring an anti-inflammatory landscape, while wild-type graft-immune cells were enriched by oxidative phosphorylation markers [20,47].

There are several limitations of our study. First, this is a single-center clinical investigation and includes a relatively small number of patients. The study included only CML patients; however, there is a rational need to explore the role of RNLS in Ph(-) MPNs and bone marrow fibrosis cases, given the RNLS anti-fibrotic effects. We did not perform any in vitro or in vivo experiments, which undoubtedly need to be implemented to delineate the pathophysiological mechanisms and relevance of RNLS in CML.

Our research provides new information in the field of hematological malignancies. The relevance of our study is reflected in the confirmation that RNLS is altered in this malignancy as well. However, unlike malignancies of another nature, RNLS plasma levels are significantly decreased. Treatment-naïve CML patients display lower values than those on TKI treatment, suggesting that RNLS alteration is sensitive to modulation by TKI.

Chronic myeloid leukemia is a dynamic and complex disease with pathogenic inputs coming from the oncogene, microenvironment, metabolism, miRNA, etc. The functions of RNLS, as a multifaced molecule, may perhaps be exploited altogether in this intertwined nature of CML. It can be seen as one of the central elements whose modulation may affect several of these routes at the same time. In future perspectives, RNLS can be employed in diagnostic, prognostic, or even therapeutic options.

We aim to further analyze the discriminatory ability of RNLS compared to other CML-related cytokines and parameters of current scoring systems with a preference for testing a more personalized approach that would improve patient risk stratification and prediction of response to treatment.

Taken together, RNLS should be considered in the analysis of BCR-ABL1-related mechanisms in the development and maintenance of CML, as well as management strategies that aim to overcome TKI resistance and CML progression.

## 5. Conclusions

Our results unveil significantly dysregulated RNLS levels in chronic phase CML patients, with markedly decreased levels in TKI naïve patients compared to those in remission on TKI treatment and compared to healthy controls, thereby offering a new viewpoint on hematological malignancies. Renalase blood concentrations should be interpreted within the context of a pathological condition and the presence of inflammation. Given the implications of RNLS in cancers and our results, it is rational to pursue additional investigations that can contribute to understanding RNLS’s role in CML pathogenesis and its potential for employment in precision medicine in hematological malignancies.

## Figures and Tables

**Table 1:** Baseline characteristics of the study’s patients with complete blood count (CBC).

	CML No Treatment	CML with TKI	Controls
Age (years)	58.5 ± 19.2	59.2 ± 12.6	55.7 ± 11.1
Gender, male (n (%))	11 (55%)	24 (43.6%)	10 (50%)
Sokal score			-
low	7 (35%)	17 (31%)	-
intermediate	12 (60%)	38 (69%)	-
high	1 (5%)	0	-
WBC (×10^9^/L)	25.00 ± 99.90 *^,†^	7.50 ± 5.20	5.72 ± 0.87
NEU (×10^9^/L)	17.05 ± 58.21 *	4.40 ± 5.78	3.05 ± 0.85
LYM (×10^9^/L)	6.30 ± 14.61 *	2.70 ± 1.81	2.20 ± 0.21
BAS (×10^9^/L)	0.56 ± 3.81	0.14 ± 1.89	0.61 ± 0.73
PLT (×10^9^/L)	376.66 ± 258.09 *^,†^	294.36 ± 204.00 ^†^	204.75 ± 25.15
RBC (×10^12^/L)	3.29 ± 0.93 *^,†^	4.32 ± 0.92 ^†^	4.45 ± 0.46
HGB (g/L)	114.50 ± 27.73	128.15 ± 24.49	133.17 ± 12.90
CRP (mg/L)	4.00 ± 7.35 ^†^	3.10 ± 3.18 ^†^	0.12 ± 0.06

Results are expressed as percentages, mean ± SD, or median ± IQR. * Significant results, *p* < 0.05 between CML groups. ^†^ *p* < 0.05 vs. control group. WBC—leukocytes, NEU—neutrophils, LYM—lymphocytes, BAS—basophils, PLT—platelets, RBC—erythrocytes, HGB—hemoglobin, CRP—C-reactive protein.

## Data Availability

Data are unavailable due to patients’ privacy. The data that support the findings of this study are available on reasonable request from the corresponding author, J.M.
